# Positive Balance Recovery in Ischemic Post-Stroke Patients with Delayed Access to Physical Therapy

**DOI:** 10.1155/2020/9153174

**Published:** 2020-01-24

**Authors:** Anselmo de Athayde Costa e Silva, Alex Tadeu Viana da Cruz Júnior, Nathalya Ingrid Cardoso do Nascimento, Skarleth Raissa Andrade Candeira, Aline do Socorro Soares Cardoso Almeida, Ketlin Jaquelline Santana de Castro, Ramon Costa de Lima, Tatiana Generoso Campos Pinho Barroso, Givago da Silva Souza, Bianca Callegari

**Affiliations:** ^1^Faculty of Physical Education, Federal University of Pará, Castanhal, Pará, Brazil; ^2^Laboratory of Human Motricity Studies, Institute of Health Sciences, Federal University of Pará, Av, Generalíssimo Deodoro 01, Belém 66073-000, Pará, Brazil; ^3^Institute of Biological Sciences, Federal University of Pará, Rua Augusto Correa 01, Belém 66075-110, Pará, Brazil; ^4^Núcleo de Medicina Tropical, Universidade Federal do Pará, Belém, Pará, Brazil

## Abstract

**Background:**

Since patient's prognosis after stroke depends on its severity, brain location, and type early intervention is strongly recommended.

**Objective:**

We aimed to determine whether it is still possible to improve balance in chronic patients, who suffered Intracerebral Hemorrhagic Strokes (ICHS) or Ischemic Strokes (IS), after later intervention.

**Methods:**

34 patients who had unilateral ICHS or IS and involved the motor cortex or sub-cortical areas took part in the study. The patients underwent clinical balance evaluation (using the Berg Balance Scale) and posturographic assessment (with a capacitive pressure platform) at the time of admission to the physiotherapy and at the end of the study. The physiotherapy intervention consisted of 20 sessions of 60 minutes carried out 3 times per week, following standard protocols: stretching; passive range of motion (ROM); active assistive ROM; active ROM; resistance training; coordination and balance activities while sitting and standing, and Large-muscle activities such as walking, treadmill, stationary cycle, combined arm-leg ergometry, arm ergometry, seated stepper and circuit training.

**Results:**

In the posturographic assessment, the IS group had significant lower amplitude of center of pressure (COP) anteroposterior displacement, after physical therapy intervention. Also, the 95% confidence ellipse area of the COP and the total COP displacement showed significant interaction between the subtype of stroke and the assessment period, meaning the IS group improved their balance after treatment on the contrary of ICHS. The structural analysis of the COP reinforced these results. On the other hand, no difference was observed in the clinical scale, between the assessment periods, for any subtype of stroke.

**Conclusion:**

Only IS patients have shown balance improvements after conventional intervention. COP measurements are more sensible to assess balance in chronic patients than Berg Balance Scale.

## 1. Background

Stroke represents the fourth cause of death worldwide [1] and survivors present a combination of muscle weakness or imbalance, decreased postural control, muscle spasticity, poor voluntary control, and body misalignments [2]. Moreover, these events implicate the poor recovery of functional ability and an increased risk of falls [3]. The consequences of a stroke depend on its severity, brain location, and type. Patients prognosis is highly dependent on some baseline characteristics such as age, gender, or stroke severity [4, 5].

The cerebral accidents can be classified into Ischemic Strokes (IS), which comprises 87% of the cases and are caused by thrombotic occlusion of arteries and veins; or Intracerebral Hemorrhagic Strokes (ICHS), which comprise 13% of the cases and are caused by rupture of the vessels by hypertension or aneurysm, trauma, and penetrating cerebral injuries [6]. Although ICHS is less prevalent, it is five times more fatal than IS [6].

Balance function is necessary to most stroke patients for independent living in the community. Several studies have been conducted to correct and control balance problems [7, 8], and recently literature has aimed to establish a predictive outcome pattern of recovering, depending on the patient's characteristics, including postural control [9].

Physical rehabilitation remains the first-line intervention strategy to attenuate chronic balance impairments since it promotes brain organization and plasticity after stroke [10]. But, it is strongly recommended that the intervention begins as earlier as possible (i.e., during hospitalization) [11]. In developing countries, the public rehabilitation system is under high demand and has limited infrastructure. The patients wait for long periods without treatment and the recommended early rehabilitation does not occur. There is evidence of better recovery patterns for patients in developed countries compared to developing countries, where patients often become chronic and worse, compared to patients who get the recommended ideal scenario of an early intervention [4].

As stroke is a long-term condition with long-term needs, it is important to quantify the impact of differing stroke subtypes and also to identify important prognostic variables. Also, the effects of time after stroke (chronicity) associated to delayed access to rehabilitation on patients' follow-up have not been thoroughly investigated. In this study, we aimed to determine whether it is still possible to improve balance scores in chronic patients after later intervention. Thus, we expected that due to the long-time post-stroke and delayed access to treatment, balance impairment is set in a way that is not responsive to the conventional intervention. We further hypothesized that among these chronic patients, those that had hemorrhagic stroke could show worse results compared with subjects affected by ischemic stroke when receiving the same physical therapy protocol.

## 2. Methods

This is an observational, descriptive, and analytical study that evaluated stroke patient cases referred for rehabilitation from the public health system. The treatment took place at the Demetrius Medrado Reference Center and Unit of Education and Care in Physical Therapy at the State University of Pará, Belém, Pará, Brazil.

### 2.1. Participants

Stroke participants were recruited based on the following criteria: the participants had experienced a unilateral stroke that was hemorrhagic or ischemic and that involved the motor cortex or sub-cortical areas. They were enrolled if they had lower extremity Brunnstrom stage [12] between 3 and 4, and were able to undertake independent walking, defined as the ability to walk ten meters without any assistance (supervision, orthoses or assistive devices). They were not under any physical therapy and/or exercise program prior to the enrolment in the study. Additionally, they should present an average delay of physiotherapy after the stroke event from at least 18 months. Participants were also excluded if they were unable to follow verbal requests, had other neurological, musculoskeletal, or orthopedic conditions, or were on antispastic medication at the time of the study (including botulinum toxin injection before physical therapy).

All participants gave their written informed consent to participate in the study. This study has been approved by the internal ethics committee of the Federal University of Pará (report #141.605).

### 2.2. Procedures

#### 2.2.1. Primary Outcomes: Demographic, Clinical and Functional Assessments

All patients underwent an initial anamneses and clinical examination by an experienced physiotherapist, prior to any experimental procedure. Participants had their demographic data and stroke characteristic collected in the anamneses and were then evaluated using the Berg Balance Scale (BBS) to assess dynamic balance ability. The BBS consists of 14 items and is used in clinical practice to assess hemiplegic patients' balance when walking and standing, Each task of the BBS is rated on a five-point scale (0–4) with the maximum score of 56 indicating good balance [13].

All evaluations were performed at the time of admission to the physiotherapy (pre-physiotherapy/assessment period1) and after the treatment (postphysiotherapy/assessment period 2). The physiotherapy intervention consisted of 20 sessions of 60 minutes carried out following standard protocols (Table 1) without any intervention from the researchers. It was performed 3 times per week, and although the treatment was similar for all subjects, they participated individually in their sessions. Exercises were performed in a series of ten repetitions, or 5 minutes (with 1 minutes rest period), and all subjects complied with the treatment protocol, which is summarized in Table 1.

#### 2.2.2. Secondary Outcomes: Posturography

Balance measurements were performed using the capacitive pressure platform EPS/R1, with 2224 sensors distributed in 48 cm^2^ and connected to a computer with Biomech software (Loran Engineering, Castel Maggiore, Bologna, Italy). Environmental illumination and sound conditions were kept constant during the evaluation of all subjects. The static analyses were performed with the individuals standing barefoot, with their feet apart at a distance proportional to the shoulders distance and their arms lying along the body. The subjects directed their gaze to a white circle target painted on the wall, 1 m away. Variables were recorded in 3 sessions of 1 min each, with “open eyes” condition, to prevent falls or injuries during all testing, an investigator stood close to each participant. A 60 s rest interval separated 2 consecutive recording sessions. For further data analyses, the mean values calculated from the 3 sessions recorded were used.

#### 2.2.3. Computation of Center of Pressure (COP) Parameters

The data from the platform were sampled at 100 Hz. All signals were filtered with a 35-Hz low-pass second-order Butterworth filter and converted into COP data using Biomech software (Loran Engineering, Castel Maggiore, Bologna, Italy), which was compiled with MATLAB routines (The Mathworks, Natick, MA). Conventional stabilographic analysis of COP data led to the calculation of the parameters: (1) 95% confidence ellipse area of the COP sway (COP*_area_*, in cm^2^), (2) COP excursion (defined as the maximum displacement of the COP in each direction, and globally (COP*_ap_*, COP*_ml_* and COP*_dist_*) and (3) mean velocity (M*_veloc_* in cm/s).

We also computed the parameters of a structural analysis of the COP as previously proposed by several authors [14, 15]. The sway density curve was built and is characterized by peaks that represent instants of time in which the moment of force in the ankle and the motor commands are relatively stable and by valleys that represent the instants of time in which the moment of force in the ankle. The following additional variables were measured: mean time interval between successive peaks (MT, in seconds), mean value of the peaks (MP, in seconds) and mean distance between successive peaks (MD, in millimeters).

### 2.3. Statistical Analysis

All statistical analyses were performed in R v.3.2.3 (R Corte Team 2015) with R Studio v. 0.99.893 (Rstudio Team 2015). Data were presented in mean and ±standard deviation. Normality was assessed by Shapiro-Wilk test. The comparison between assessment periods (pre- and post-physiotherapy) and type of stroke (ICHS or IS) was carried out using a two-way ANOVA. Statistical significance level was set in *p* ≤ 0.05.

## 3. Results

Thirty-four patients with a mean age 55.5 ± 11.4 years old (54.2 ± 11.9 women, *n* = 11 and; 56.1 ± 11.3 men, *n* = 23) participated in the study. The mean weight of the participants was 62.2 kg (±13 kg) and mean height was 164 cm (±12 cm).

The baseline characteristics assessed were age, sex, time since last stroke onset episode, stroke type, and affected body side. 35% of the 34 patients were affected by ICHS and 65% by IS. The right hemisphere of the brain was affected in 62.5% of the patients and the remaining 37.5% was affected in the left hemisphere. The average delay of physiotherapy after the stroke event was 52.8 months (±41.7 months) for IS and 54.0 months (±42.9 months) for ICHS. The total average number of subsequent stroke episodes was 1.55 (±0.78).

Comparison between the pre- and postphysiotherapy stage are summarized in Table 2. No difference was observed in BBS scale, between the assessment periods, for any subtype of stroke. Also, no interaction between assessment periods and type was observed for this variable (*F* = 0.68, *p* = 0.41).

**Figure 1:**
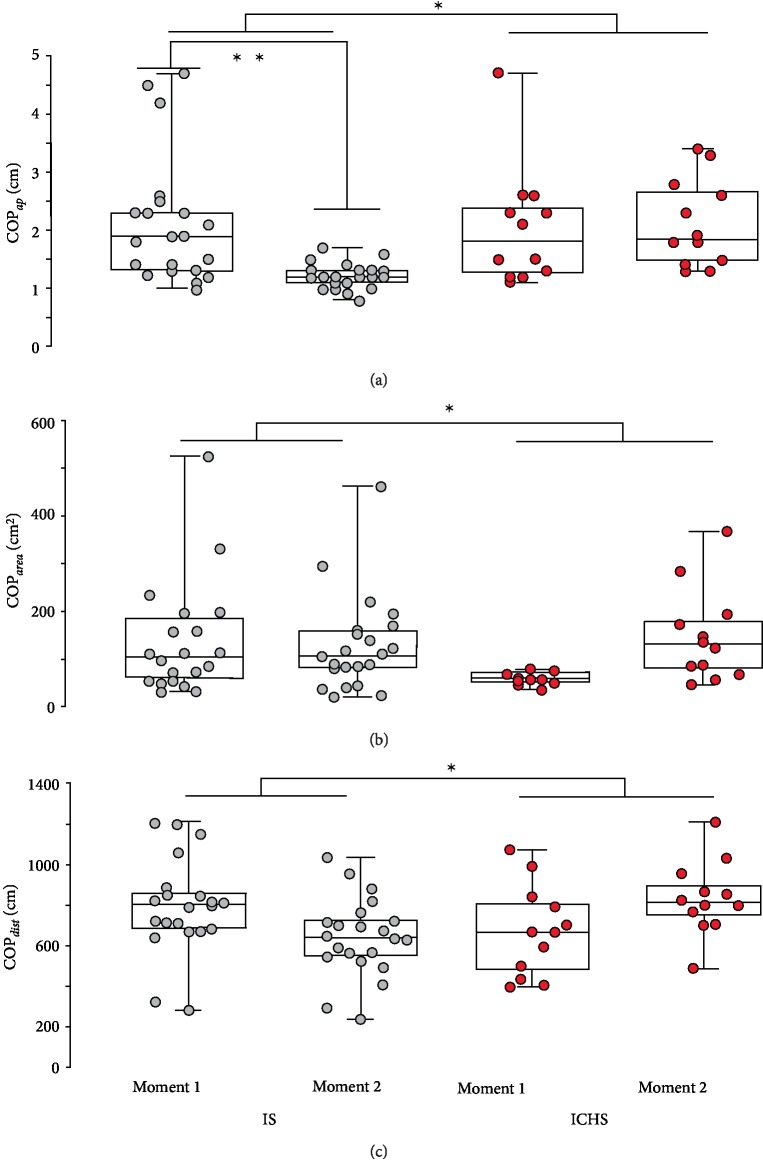
Conventional stabilographic analysis of COP parameters. Boxplots plot with 95% Confidence Interval showing the assessment periods 1 pre- and 2 postphysiotherapy to IS and ICHS patients. (a) COP*_ap_*; (b) COP*_area_*; (c) COP*_dist_*. ^∗^Significant type/assessment periods interaction. ^∗∗^Significant difference to pre.

On the other hand, the posturographic parameters showed interesting behavior that differed between stroke subtypes. Considering COP*_ap_*, a significant assessment periods effect (*F* = 29.63, *p* < 0.001) and interaction between the subtype and the assessment periods were observed (*F* = 0.01, *p* < 0.001, Figure 1). No differences were observed in mediolateral displacement, measured by COP*_ml_* (*F* = 0.06, *p* = 0.901). Thus, IS group had significant lower amplitude of anteroposterior displacement, but not mediolateral displacement. Analyzing COP*_dist_* and COP*_area_*, significant interaction was observed between the subtype of stroke and the assessment periods for both variables. These posturographic parameters showed that the IS group improved their balance after treatment on the contrary of ICHS (COP*_dist_*: *F* = 8.911, *p* = 0.004 and COP*_area_*: *F* = 8.33, *p* = 0.005, Figure 1).

**Figure 2:**
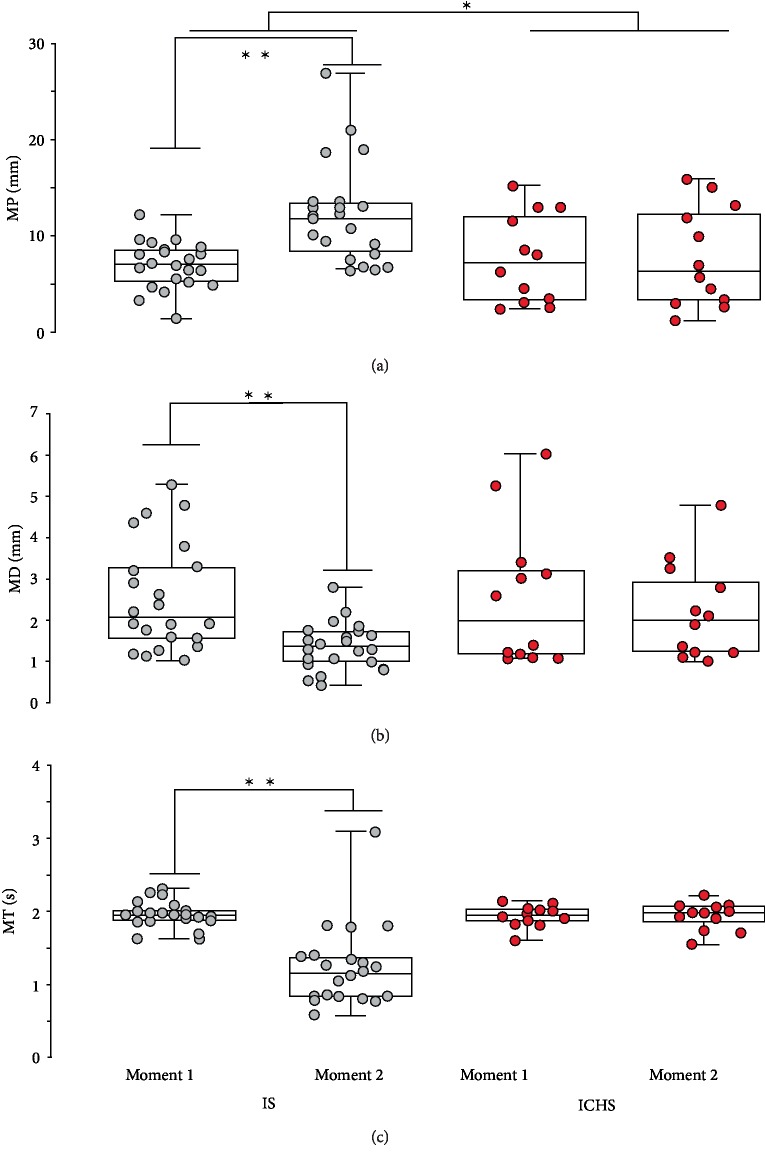
Structural analysis of COP parameters. Boxplots plot with 95% Confidence Interval showing the assessment periods 1 pre- and 2 postphysiotherapy to IS and ICHS patients. (a) MP, (b) MD, and (c) MT. ^∗^Significant type/assessment periods interaction. ^∗∗^Significant difference to pre.

Variables from structural analysis of the COP reinforce these results as significant assessment periods effect was observed for MP (*F* = 10.7, *p* = 0.001), MD (*F* = 9.268, *p* = 0.003) and MT (*F* = 32.2, *p* < 0.001). Also, significant interaction between the subtype of stroke and the assessment periods was also positive observed for MP (*F* = 5.34, *p* = 0.005) and MT (*F* = 17.4, *p* < 0.001). As depicted in Figures 1 and 2, IS had greater mean peaks after treatment, and slower time interval and distance between one peak and the subsequent, what represent better stability.

## 4. Discussion

The main objective of our study was to analyze if it is still possible to improve balance in chronic patients, years after stroke. We set this objective because in developing countries the late intervention is a common, unhappy reality. Early intervention is preconized and especially for patients with hemorrhagic stroke, long waiting for the intervention is disadvantageous, due the severity of damage compared to ischemic patients. Here, we found that only the ischemic patients had improvements on balance. In addition, balance improvements were only evident with posturographic assessment. This indicates that this common balance scale is less sensible to such changes.

### 4.1. IS versus ICHS Stroke Recovery

We found that subjects with hemorrhagic damage had worse condition to respond to the rehabilitation program. Indeed, the type of stroke is highly related to the severity of the impairments and mortality [16]. While IS is more common (accounts for about 87 percent of all strokes) and leads to a loss of neurologic function dependent to the extension of the brain area that the obstructed vessel supplies, ICHS is a stroke subtype that is associated with high mortality (~40% at one month) and those that survive often have major neurological impairments [16]. This may reflect differences in spontaneous recovery and may explain the improvements we observed in our chronic ischemic patients. Comparisons between ICSH and IS severity, mortality, and risk factors, found in the literature, also supports our results. Approximately 10% of 4000 stroke patients had ICHS and the stroke severity was almost linearly related to the hemorrhagic nature of the lesion [17]. In addition, lesion sizes in these patients were larger than in patients with IS. In a Copenhagen Stroke Study [18], authors showed that the largest diameter of lesions in patients with ICHS was by 20% larger compared to IS patients.

### 4.2. Intervention Period

Our patients were admitted with a delay of approximately 50 months to physiotherapy, far later than the stroke event. Evidence from clinical trials supports the premise that early initiation of therapy improves recovery from stroke due to greater gains and benefits for neuroplasticity and in functionality, especially in more severe cases [9, 19]. In this period happens an early spontaneous improvement that includes the resolution of local edema, resorption of local toxins, improvement of local circulation, and recovery of partially damaged ischemic neurons [20].

Some studies have suggested that stroke rehabilitation is most effective and improves functional outcomes if initiated even early (within three to 30 days post stroke) [21, 22]. Paolucci et al. [23] assessed the specific influence of the onset-admission interval on rehabilitation results in 145 patients and found that beginning treatment within the first 20 days had significantly higher effectiveness of treatment than later periods. Agreeing with them. Bai et al. [24] concluded that early rehabilitation can significantly improve the daily activities and motor functions of patients, even in intracerebral hemorrhagic stroke.

### 4.3. Types of Rehabilitation Therapy

Beyond the stroke type and the beginning of the intervention, another factor that can influence recovery post stroke is the type of physical therapy [25]. In part, we observed that the conventional rehabilitation was not effective in late interventions, especially for ISCH. Actually, recent literature in general, often associates the benefits for chronic ICHS and IS patients to the use of non-conventional physical therapy protocols [3, 8, 21].

Uçar et al. [26], for example, tested the feasibility and potential efficacy of using a robotic-assisted gait device for treadmill training with partial body weight support in subjects with chronic IS or ICHS patients (at least 12* *months of lesion). They suggested that the robotic-assisted device group demonstrated significantly greater improvement in gait assessments than the group undergoing conventional physical therapy. Results from another randomized, controlled study indicated that even long after a stroke, kinesthetic ability training, administered in combination with a conventional rehabilitation program, can improve balance in hemiparetic stroke patients [27].

### 4.4. Assessment Methods

In our study we only observed improvements in the posturographic assessments with no significant results in the clinical scale. There are several assessment tools to measure balance function, including the Berg Balance Scale, which is the most commonly used assessment tool across the continuum of stroke rehabilitation. However, its effectiveness is discussed since it is indicated that the BBS often fails to detect differences in balance at the high end of the scale. Recent investigation found that the lowest BBS score was above 44, which, in turn, limited the ability of the BBS to discriminate among subjects with spinal cord injury. This fact that did not occur with the posturographic assessment, which was reliable, valid, and effective in acquiring data [28]. This may explain our results, where in BBS, the differences according to the different stroke subtypes were not perceived, while COP parameters showed best recovery in ischemic patients. A recent systematic review and meta-analysis [29] supports that and in spite of the several papers reporting predictive validity of the BBS to detect fall risk, authors states that other more objective evaluation methods can be implemented by analyzing center of pressure (COP) parameters using stabilometric platforms (SPs). In general, when comparing postural responses exhibited by poststroke patients, authors found different COP responses to perturbation in two groups despite having similar BBS scores. Finally, they suggested the need of a different assessment protocol for testing balance in high-functioning patients.

### 4.5. Limitation and Practical Applications

Our study has some potential limitations. First, we used only one clinical scale to assess physical therapy improvements. It is possible that the lack of sensibility of the scale compared to the posturography may hide the improvements. On the other hand, we have used the posturography, one reliable measurement to evaluate balance improvements. But we only assessed in static conditions. Maybe it would be interesting to perform the same investigation in dynamic conditions, as adopting a more ecological model may help to determine a mechanism for improving balance control.

Despite these limitations, the use of the BBS and posturography deserves further comments. We did not observe significant improvements in BBS, as we did in the posturography. One can ask if the difference observed in the posturography may have clinical relevance, since the BBS measures both static and dynamic aspects of balance and is more related to the daily activities. This is a functional scale, but individuals may continue to recover neurologically and achieve higher levels of functional status, and this scale may not be sensitive to these changes. This is the main reason why we performed the posturography measurements before and after delayed physiotherapy treatment of stroke patients.

We believe that our results can be useful for clinicians who are often requested to predict outcome after stroke by the patient, family, other healthcare workers, and insurance providers. In order to advocate for the most appropriate rehabilitation programs for stroke patients, health professionals must understand patterns of recovery and know the limitations of existing studies.

## 5. Conclusion

Only patients with ischemic damage have shown balance improvements after the adopted intervention. Apparently, the late rehabilitation has less beneficial effects in hemorrhagic damaged patients. Due to the differences in sensitivity between Berg Balance Scale and COP measurements, we suggest that the COP are more sensible to assess balance in chronic patients and may be useful in decision regarding the duration of rehabilitation, selection of interventions, and discharge planning.

## Figures and Tables

**Table 1:** Rehabilitation protocol.

Intervention	
(i)	Stretching; passive range of motion (ROM)
(ii)	Active assistive ROM; active ROM
(iii)	Resistance training: isometric exercise; free weights, weight machines
(iv)	Functional electrical stimulation of the upper and lower limb which practicing functional tasks
(v)	Coordination and balance activities while sitting and standing
(vi)	Large-muscle activities such as walking, treadmill, stationary cycle, combined arm-leg ergometry, arm ergometry, seated stepper; circuit training
Major balance goal	

(i)	Increase ROM and flexibility of lower extremities
(ii)	Increase strength and improve muscular endurance in lower limb muscles
(iii)	Increase core or trunk musculature strength;
(iv)	Maintain joint range and alignment
(v)	Maintain static standing balance with feet shoulder-width apart, minimizing the assist
(vi)	Improve transitions from one posture to another (i.e. sit to stand), minimizing the assist
(vii)	Improve shifting weight forward/backward and between sides, minimizing the assist
(viii)	Help prevent falls

Standardized rehabilitation protocol implemented by physiotherapists at public treatment centers.

**Table 2:** Mean and standard deviation of BBS and COP measures, pre- and postrehabilitation.

Outcomes	Prephysiotherapy (assessment periods 1)		Postphysiotherapy (assessment periods 2)	
	IS	ICHS	IS	ICHS
BBS*_scale_* (points)	49.9 (±9.4)	46.1 (±7.8)	47.3 (±7.8)	44.8 (±10.1)
COP*_dist_* (cm)	797.0 (±230.6)	693.9 (±222.8)	641.9 (±192.1)	835.4 ± (180.3)^∗^
COP*_area_* (cm^2^)	136.9 (±115.1)	60.9 (±13.2)	102.0 (±54.2)	148.0 (±96.4)^∗^
COP*_ap_* (cm)	2.1 (±1.1)	2.0 (±1.0)	1.6 (±1.7)^∗∗^	2.1 (±0.8)^∗^
COP*_ml_* (cm)	2.6 (±1.0)	2.4 (±1.5)	2.6 (±1.4)	2.6 (±1.8)
M*_veloc_* (cm/s)	14.8 (±3.7)	10.9 (±2.7)^#^	11.3 (±3.5)^∗∗^	10.7 (±3.0)^∗^
MT (sec)	2.0 (±0.2)	1.9 (±0.1)	1.2 (±0.6)^∗∗^	1.9 (±0.2)
MP (mm)	6.9 (±2.4)	7.6 (±4.6)	12.3 (±5.2)^∗∗^	7.8 (±5.2)^∗^
MD (mm)	2.6 (±1.3)	2.5 (±1.7)	1.4 (±0.6)^∗∗^	2.2 (±1.9)

Values represent mean and standard deviation, IS (ischemic stroke), ICHS (Intracerebral Hemorragic Stroke) BBS (Berg Balance scale), COP*_area_* (ellipse area of the COP sway), COP*_dist_* (total COP excursion), COP*_ap_* (anteroposterior COP displacement), COP*ml* (medium-lateral COP displacement), M*_veloc_* (COPmean velocity), MT (mean time interval), MP (mean value of the peaks) and MD (mean distance). ^∗^Significant type/assessment periods interaction. ^∗∗^Significant difference to pre. ^#^Significant difference between types of stroke.

## Data Availability

The datasets used and/or analyzed during the current study are available from the corresponding author on reasonable request.
